# Artificial Intelligence-Based Treatment Decisions: A New Era for NSCLC

**DOI:** 10.3390/cancers16040831

**Published:** 2024-02-19

**Authors:** Oraianthi Fiste, Ioannis Gkiozos, Andriani Charpidou, Nikolaos K. Syrigos

**Affiliations:** Oncology Unit, Third Department of Internal Medicine and Laboratory, Medical School, National and Kapodistrian University of Athens, 11527 Athens, Greece; yiannisgk@hotmail.com (I.G.); dcharpidou@yahoo.gr (A.C.); nksyrigos@gmail.com (N.K.S.)

**Keywords:** artificial intelligence, AI, machine learning, big data, radiomics, pathomics, treatment, biomarkers, non-small cell lung cancer, lung cancer

## Abstract

**Simple Summary:**

Lung cancer therapeutics have dramatically improved in recent years. Indeed, precision oncology could be exemplified by non-small cell lung cancer (NSCLC), with molecular profiling and programmed death ligand 1 (PD-L1) immunohistochemical expression representing an integral part of its tailored treatment. The present narrative review aims to highlight the promising role of artificial intelligence (AI) technologies in the optimal, patient-centered management of NSCLC, by distilling as well as interpreting big data.

**Abstract:**

Non-small cell lung cancer (NSCLC) is the leading cause of cancer-related mortality among women and men, in developed countries, despite the public health interventions including tobacco-free campaigns, screening and early detection methods, recent therapeutic advances, and ongoing intense research on novel antineoplastic modalities. Targeting oncogenic driver mutations and immune checkpoint inhibition has indeed revolutionized NSCLC treatment, yet there still remains the unmet need for robust and standardized predictive biomarkers to accurately inform clinical decisions. Artificial intelligence (AI) represents the computer-based science concerned with large datasets for complex problem-solving. Its concept has brought a paradigm shift in oncology considering its immense potential for improved diagnosis, treatment guidance, and prognosis. In this review, we present the current state of AI-driven applications on NSCLC management, with a particular focus on radiomics and pathomics, and critically discuss both the existing limitations and future directions in this field. The thoracic oncology community should not be discouraged by the likely long road of AI implementation into daily clinical practice, as its transformative impact on personalized treatment approaches is undeniable.

## 1. Introduction

Lung cancer (LC) represents an aggressive malignancy of significant prevalence, worldwide, with an estimated 238,340 new cases and 127,070 deaths in 2023 in the United States (US) alone [1]. Small cell LC (SCLC) and non-small cell LC (NSCLC) are the main histological subtypes, with the latter being the most common and further being classified into adenocarcinoma (adNSCLC), squamous cell carcinoma (sqNSCLC), and large cell carcinoma (LCLC) [2]. Global disparities in both LC incidence and mortality have been acknowledged, reflecting health inequities, smoking divergences, varying patterns of environmental exposure, and genetic factors [3]. Historically, LC rates were predominantly higher in older male smokers, yet recent epidemiological data suggest not only a continuously increasing female-to-male incidence rate ratio [4], exceeding 1.0 in the younger age group of 30–49 years [5], but also a quite significant proportion of non-smoking LC cases varying between 10 and 16% for men and women, respectively [6,7].

Hence, the rather strict eligibility criteria of certain high-risk individuals (heavy smoking history in adults aged 50 and over in particular) for annual low-dose computed tomography (LDCT) screening may need to be modified [8]. Moreover, despite robust clinical evidence of LDCT efficiency [9,10], its low uptake rates could, at least partially, explain the rather delayed diagnoses [11,12,13]. Nevertheless, major advances in LC therapeutics, including targeted therapy in patients with an actionable driver mutation or antibody-directed immunotherapy against the specific checkpoint molecules programmed death-1 (PD-1), its ligand (PD-L1), and the cytotoxic T-lymphocyte-associated protein 4 receptor (CTLA-4), with or without classic cytotoxic chemotherapy, have undoubtedly contributed to survival improvements during the last decade [14]. It is, therefore, of paramount importance to accurately identify those patients who are most or least likely to derive benefit from these novel therapies.

Currently, according to the European Society for Medical Oncology (ESMO) and the National Comprehensive Cancer Network (NCCN) guidelines, comprehensive molecular testing via high-throughput next-generation sequencing (NGS) is recommended in every patient with advanced non-squamous carcinomas and in unusual cases of sqNSCLC (i.e., younger than 50 years of age, never- or former light- or long-time ex-smokers), as oncogene-addicted metastatic NSCLC epitomizes a prominent example of ‘precision oncology’, whereas PD-L1 immunohistochemistry (IHC) testing remains the solely established predictive biomarker for immune checkpoint inhibition (ICI) [15,16]. Focal tissue sampling, however, fails to assess both the spatial and temporal tumor heterogeneity [17], while diverse prognosis to epidermal growth factor receptor (EGFR)-tyrosine kinase inhibitors (TKIs) highlights the necessity of EGFR genotype stratification [18,19]. Of note, *EGFR* gene mutations were the first to be targeted and are found in about 15–32% of NSCLC cases [20,21]. PD-L1 expression is also characterized by intra- and inter-tumoral heterogeneity [22], whilst inter-assay discordances among several approved companion diagnostic tests make PD-L1 positivity interpretation challenging [23].

Artificial intelligence (AI), an expanding branch of computer science for human intelligence augmentation, has emerged as a transformative force in oncology [24]. In fact, Luchini et al. have reported that, by the end of 2021, 71 AI-enabled medical devices have been authorized by the United States Food and Drug Administration (FDA) within several oncological settings, mainly in radiology and pathology (54.9% and 19.7%, respectively), and mostly for breast malignancies (31%) [25]. While, just a few months ago, ISM3091, a novel highly selective small molecule inhibitor of ubiquitin-specific peptidase 1 (USP1), became the first AI-engineered targeted therapy to be entered in a phase 1 trial in patients with advanced, homologous recombination deficient (HRD) solid tumors (NCT05932862) [26]. The scope of this review is to present the concept of AI integration into NSCLC treatment in order to further optimize patient care and to discuss limitations as well as future perspectives.

## 2. Artificial Intelligence (AI)

AI refers to an umbrella term for a broad range of computational systems capable of simulating human cognitive functions like learning, perception, reasoning, and problem-solving, independently [27]. Its subfield, machine learning (ML), focuses on algorithmic methods development based on datasets, without explicit programming and with self-adjusting abilities through data and experience [28]. Four distinct and mutually exclusive learning types can be incorporated into the ML training process; supervised learning using labeled input data, unsupervised learning using unlabeled data, semi-supervised learning combining labeled and unlabeled data, and reinforcement learning within an interactive environment of reward and punishment mechanisms [28,29]. Deep learning (DL), a subset of ML algorithms, stands for artificial neural networks that mimic the complexity of human brain structure and activity and are capable of automatic representation learning [30].

Despite human interest in intelligent machines being encountered since antiquity, the modern AI theoretical foundation was first introduced in 1950 by Alan Turing [31], whereas the Dartmouth conference during the summer of 1956 has been widely considered the ‘birthplace’ workshop of AI [32]. The slow progress within the next decades, mainly due to insufficient funding, was followed by a huge resurgence of interest in the late 20th century. Since then, AI has witnessed remarkable advancements and has transformed various aspects of our daily lives, including biomedical research and healthcare [27,28].

AI holds the potential to reshape LC management, encompassing new and promising approaches for screening, early diagnosis, molecular characterization, optimized management, and accelerated drug development [33]. With regards to the specific focus of our review, AI-based technologies leverage vast data sources (i.e., patients’ clinical records, imaging data, genomics, etc.) to identify potential biomarkers of prognosis, accurate prediction of treatment efficacy, and real-time monitoring of individualized responses, permitting tailored therapeutic plan [34].

### 2.1. AI-Driven Radiomics

Imaging with CT and/or positron emission tomography/CT (PET/CT), which has traditionally been the gold standard for treatment planning in LC patients [35], could be combined with the sophisticated computational approaches of AI into radiomics models, for non-invasively extraction of subtle peri- and intra-tumoral features, providing unbiased information for LC and its microenvironment [36,37]. Apart from being non-invasive and less susceptible to tumor heterogeneity, radiomics can be reproducible and less expensive compared to tissue sampling [37]. The typical workflow of radiomics involves the following steps:

- Medical image acquisition using the proper modality (CT, PET/CT, other);
- Acquired image preprocessing, using noise reduction, image resizing, and contrast enhancement, to improve data quality;
- Segmentation of tissue in order to define the region of interest (ROI);
- Quantitative features extraction, including tumor size, shape, texture, and signal intensities, which can reflect the lesion’s malignant potential as well as its heterogeneity;
- Relevant features selection for improved subsequent analyses;
- Extracted features normalization in datasets to eliminate inconsistencies among different imaging techniques or protocols;
- AI-based model development;
- AI-based model (internal or external) validation in independent datasets to assess its performance and generalizability;
- Correlation of model predictions (radiomic features) with patients’ data (clinical outcomes); and
- Integration, validation, and refinement of validated radiomics within clinical workflows [38].

In 2020, the Image Biomarker Standardization Initiative (IBSI) reported a list of 169 radiomic features characterized by their standard definition and their proven reproducibility [39]. Furthermore, to reduce any diverging distributions within LC radiomic datasets, several statistical harmonization techniques have been proposed, with BM-ComBat providing the best performance thus far [40].

In addition, multi-region radiomics might be explored for their useful information regarding lung background. Indeed, areas affected by pulmonary fibrosis or chronic obstructive pulmonary disease, emphysema in particular, carry a higher risk of carcinogenesis, while areas of ground glass opacity tend to confer a more favorable prognosis [41,42]. Current literature also suggests that the combination of imaging features with the underlying genomic phenotypes, also known as radiogenomics, enables the refined assessment of “whole-tumor” biological complexity and the evidence-based guidance of personalized treatment [43]. A selection of representative studies regarding radiomics applications in NSCLC therapy is presented in Table 1.

Cheng et al. applied gradient boosting to differentiate the *EGFR* mutation status in a total of 1476 radiomics features using pre-operative CT images from 636 adNSCLC patients. The established ML-based model consisted of 102 features, yielding an area under the curve (AUC) of 0.8 for the external validation cohort [44]. Considering that not all *EGFR*-mutated adNSCLC patients respond equally to EGFR-TKIs, the EGFR genotype is rather crucial [18,19]. In a retrospective study, Li et al. investigated the potential of CT radiomics to predict the common *EGFR* mutations, exon19 deletion, and exon21 L858R point mutation, and reported an AUROC for the test data set of 0.79 for 19del and 0.78 for L858R [45]. Both of these respond rather well to EGFR-TKIs, yet 19del seems to correlate with better survival outcomes [63,64]. The most frequent mechanism of resistance to EGFR-targeted therapy, the acquired exon20 T790M mutation, can also be predicted via ML-driven radiomics, especially in conjunction with clinical factors [46].

Using the least absolute shrinkage and selection operator (LASSO) regression model, Yang et al. showed that the numerous radiomics features extracted from different time phases of CT imaging could not only predict *EGFR* mutation status but also assess the sensitivity to TKIs, with the venous-derived features being correlated with the best performance (AUC: 0.91) [47]. In another large study of >18,000 LC patients, the research group combined a fully automated whole-lung AI analysis with conventional CT imaging and confirmed the superiority of incorporating radiomics-based genotype into clinical data, like age, sex, stage, histology, and smoking status, for the prediction of response to EGFR-TKIs [48].

Anaplastic lymphoma kinase (ALK) aberrations have been identified, since 2007, in <7% of NSCLC cases [65], and currently several TKIs are approved for the treatment of advanced ALK+ disease [15,16]. Both CT- and PET/CT-based radiomics have successfully predicted ALK rearrangement status, with AUC of 0.89 and 0.88, respectively, but in the case of PET/CT radiomics the ML model performance was not improved when implemented both imaging and clinical data [49,50]. Among the studies regarding comprehensive genotyping, Shao et al. used a DL methodology to evaluate not only a panel of oncogenic driver mutations but also the IHC assessment of PD-L1, reflecting the realistic daily clinical practice, with varying AUC of 0.796–0.912 [51].

Various CT- and/or PET/CT-derived radiomics have been utilized to predict PD-L1 expression levels [52,53,54,55,56]. Despite being single-center and of retrospective design, the study by Wang et al. used a DL algorithm based on CT radiomics and reported robust, high AUC scores of 0.95, 0.934, and 0.946 for PD-L1 tumor proportion score (TPS) < 1%, 1–49%, and ≥50%, respectively [54]. Similarly promising results have been achieved in a cohort of 399 NSCLC patients with an ML methodology using CT- and PET/CT radiomics; CT radiomics outperformed selected features from other imaging modalities in both PD-L1 TPS > 1% (AUC: 0.97) and TPS > 50% (AUC: 0.80) [56].

Regarding treatment response, Trebeschi et al. evaluated the gene set enrichment analysis computational method in a cohort of 385 patients with advanced-stage NSCLC, under anti-PD-1 immunotherapy and showed that CT radiomics could provide predictive biomarkers in a non-invasive way [57]. Valuable information in regard to treatment response could be provided in earlier stages too; Ramella et al. developed an ML-based radiomics model to predict the therapeutic efficacy of concurrent chemoradiation (cCRT) in 91 stage III NSCLC patients, yielding quite satisfactory results (AUC: 0.82) [59].

AI can also play a crucial role in tumor microenvironment (TME) analysis [36,37]. TME, comprising of tumor cells, infiltrating immune cells (i.e., neutrophils, macrophages, T- and B- lymphocytes, etc.), stroma cells, chemokines, and other cellular and non-cellular components, determines disease aggressiveness and therapeutic response, thus highly influences clinical outcome [66,67,68,69]. In-depth characterization of the TME landscape using AI-based approaches in combination with single-cell technology could unveil novel predictive biomarkers for optimized treatment decisions. Indicatively, Sun et al. developed an AI model based on CT radiomics in order to predict CD8 infiltration and its predictive value of response to ICI, with rather modest results (AUC of 0.63–0.76) [60,61]. On the contrary, an ML-based PET/CT radiomics signature successfully identified TME features predictive of immunotherapy response in a cohort of 194 patients with locally advanced and metastatic NSCLC [62].

### 2.2. AI-Driven Pathomics

Tissue biopsy specimen analysis remains the cornerstone of definitive diagnosis and comprehensive molecular analysis of cancer, including NSCLC. The integration of multi-omics (genomics, transcriptomics, proteomics, and metabolomics) into histopathology datasets could provide the missing information on structural–morphological tissue changes in disease [70]. AI-driven technologies can be used to analyze the vast amount of these complex pathomics data for improved diagnostic, prognostic, predictive, and stratification purposes [71,72]. Moreover, the digitalization of traditional pathological tissue slides using whole-slide imaging (WSI), for clinical, research, and educational initiatives [73], subsequently led to the development of such computer-based algorithms [74].

AI-driven pathomics workflow complements a quite comparable approach used in radiomics [75]. To the best of our knowledge, contrary to the latter, AI-assisted pathomics studies in NSCLC are limited. Table 2. summarizes the reviewed literature regarding pathomics-based technologies in NSCLC treatment guidance.

Coudray et al. trained a DL algorithm on >1000 WSI from the Cancer Genome Atlas (TCGA) for further histological classification of NSCLC cases and accurate prediction of their mutational status. The AI-based pathomics model successfully discriminated LC from normal tissues (AUC of 0.99), distinguished non-squamous histology from sqNSCLC cases (AUC of 0.97), and demonstrated high accuracy in molecular genotype (AUC of 0.754, 0.814, and 0.845 for *EGFR*, *KRAS*, and *STK11* mutations, respectively) [76].

A convolutional neural network (CNN), a subset of ML approaches, has been used to generate image classification for gene fusion detection. More specifically, an Israeli research institution validated such an AI-driven pathomics approach to detect *ALK* and *ROS-1* rearrangements, which were found to be highly sensitive (100%) and specific (100% and 98.6% for *ALK* and *ROS-1* fusions, respectively) [77]. The relevant issue of tissue insufficiency to continue with molecular testing has been addressed by a Chinese study, in which a CNN-based methodology not only differentiated benign from malignant pleural effusions with an AUC of 0.93 but also identified the primary tumor site (accuracy rate for adNSCLC of 0.81) and predicted gene aberrations with the usage of pleural effusion cell block WSI [78].

PD-L1 TPS IHC testing represents the sole validated biomarker of response to immunotherapy [15,16], yet its interpretation through routine histopathology reports remains subjective and semi-quantitative [83]. AI-driven computational pathology may overcome such limitations relative to the human bias of manual scoring [79,80]. Additionally, it could quantify tumor-infiltrating lymphocytes (TILs), which have been correlated with favorable prognosis and ICI efficacy [84,85]. Rakaee et al. developed an ML-based methodology to evaluate TILs and combined predictive models (TILs/PD-L1, TMB/PD-L1) in immunotherapy responders. Interestingly enough, both models outperformed PD-L1 expression assessment with regards to the ICI response (AUC of 0.77 and 0.65 for TILs/PD-L1 and TMB/PD-L1, respectively), whilst in the PD-L1 negative cohort, TILs preferably identified responders compared with TMB [79]. The authors concluded that TIL-level quantification is a cost-effective, easily implemented method that could be translated into routine clinical practice if validated in larger, prospective studies [79].

Nibid et al. showed that deep pathomics, based on ore-treatment specimens, was not only highly specific (true negative rate of 90.1) but also rather sensitive (true positive rate of 0.75) to predict responses of patients with locally advanced NSCLC treated with cCRT [81]. These results, in accordance with their previous radiomics study, underscore the capabilities of AI-driven analysis of omics for the optimal management of patients with stage III NSCLC, who are at risk of relapse [59,81].

Lastly, prognostic stratification is critical for guiding adjuvant treatment recommendations for early-stage disease. In a multicenter retrospective study, Lin et al. proposed an immune scoring system based on TME of automated assessment of cell density in NSCLC patients, who underwent upfront surgery with curative intent, which could predict disease-free survival (DFS) [82].

## 3. Discussion

As thoroughly discussed in the present review, AI integration into NSCLC management represents a continuously expanding and transformative field, utilizing data-driven, personalized strategies. The ever-growing literature accentuates the potential of AI-driven radiomics and pathomics in predicting treatment response, both directly and indirectly, using accurate predictive biomarkers like PD-L1, TME, and mutational status. However, various hurdles still exist and need to be addressed.

With regards to radiomics, feature reproducibility is of utmost importance, considering the varying image acquisition (including the highly variable CT protocols and slice thickness), preprocessing, and segmentation [86,87]. PET/CT scan undeniably provides a vast amount of imaging data and parametric information, yet it can be correlated with pitfalls and/or artifacts, while it remains more expensive and difficult from a technical requirement perspective [88]. The absence of universal consensus on the optimal threshold for LC radiomics should also be considered [89]. In the case of pathomics, differences in staining techniques, time, and definitions (terminology) of histopathological features are among the main implementation barriers [90]. Moreover, working with AI-based approaches should be considered as a rather specialized skill for which the next generation of radiologists and pathologists should be educated.

Furthermore, various distinct AI algorithms have been developed in NSCLC patient-centered studies (the majority of which were retrospective and single-center) and have been evaluated in rather small training sample sizes without external validation. Thereby, both their interpretability and generalizability are hindered [91]. We should also emphasize that clear ethical and legal frameworks from the engaged stakeholders (i.e., healthcare professionals, research institutions, patient advocacy groups, and government) are strictly required [92].

Future applications of AI for precision medicine in NSCLC may implement radiomics and liquid biopsies (circulating tumor cells and/or nucleic acids detection) into novel companion diagnostics, to provide valuable information on tumor biology, clonal evolution, disease progression, and response to treatment, in a minimally invasive, longitudinal fashion [93,94]. Last but not least, the novel AI-based delta radiomics model targets the quantitative features of imaging at different acquisition time points (most often during therapy) in order to accurately document data changes and, thus, reveal disease biological behavior [95]. Currently, only a limited number of publications evaluate delta radiomics in LC patients with regard to prognosis, *EGFR* mutation status, and response to treatment [58,95,96]. Of note, the United Kingdom (UK)-based project AIRIaL, which stands for Artificial Intelligence and Resistance Imaging in Lung Cancer, aims to develop novel predictive imaging features based on PET as well as AI-engineered biomaterials for targeted payloads of drugs directly to the resistant clones [97].

## 4. Conclusions

AI-based technologies, despite their infancy, have gained great attention within the oncology community as they could potentially foster optimal, personalized management of cancer patients. Indeed, by tackling the complexity of the highly heterogeneous NSCLC disease, AI approaches will pave the way for a paradigm shift in the field of informed, data-driven clinical decisions in the near future. Several challenges still remain, yet their prospective validation within a large number of institutions over diverse populations will ultimately lay the foundation for their real-world implementation.

## Figures and Tables

**Table 1:** Selected studies of AI-based radiomics in NSCLC treatment.

Reference	Author, Year	AI-Based Methodology	Model Features	Target Variable	Patient Population	Training Cohort	Validation Cohort	Best Performance
[44]	Cheng et al., 2022	Gradient boosting decision tree (GBDT)	CT radiomics	*EGFR* mutation	adNSCLC	N = 464	N = 172	0.838 (training set); 0.822 (internal validation set); 0.803 (external validation set)
[45]	Li et al., 2019	Logistic regression (LG)	CT radiomics	*EGFR* 19del and L858R	NSCLC	N = 236	N = 76	0.7925 (19del); 0.775 (L858R)
[46]	Yang et al., 2022	Least absolute shrinkage and selection operator (LASSO) regression model	CT radiomics	T790M mutation	EGFR-mutated adNSCLC post-progression on 1st line with 1st or 2nd generation EGFR TKI	N = 186	N = 74	0.71
[47]	Yang et al., 2020	LASSO regression model	CT radiomics	Response to EGFR-TKI	EGFR-mutated adNSCLC, clinical stage IIIB-IV, under 1st line with EGFR-TKI	N = 253	N/A	0.7268 (unenhanced phase); 0.7793 (arterial phase); 0.9104 (venous phase)
[48]	Wang et al., 2022	LASSO regression model	CT radiomics	*EGFR* genotype; Response to EGFR-TKI	NSCLC, stage I-IV from 9 cohorts (7 retrospective Chinese cohorts, The Cancer Imaging Archive cohort, and a prospective Chinese cohort)	Ν = 5645 (EGFR genotype and thick CT); N = 4782 (EGFR genotype and thin CT); N = 490 (Response to EGFR-TKI)	N = 3364 (EGFR genotype and thick CT); N = 6528 (EGFR genotype and thin CT); N = 110 (Response to EGFR-TKI)	0.755–0.770 (EGFR genotype and thick CT); 0.748–0.797 (EGFR genotype and thin CT); Genotype predicted by the fully automated AI-system (FAIS) combined with clinical factors (FAIS-C model) was significantly associated with EGFR-TKI prognosis (*p* < 0.05)
[49]	Hao et al., 2022	LASSO regression model	CT radiomics, clinical and radiographic (CT) features	*ALK* rearrangement	In situ and invasive adNSCLC, stage I-IV	N = 154	N = 39	0.914 (CT image and clinical features-based ML model); 0.89 (CT image-based ML model); 0.735 (clinical features)
[50]	Chang et al., 2021	LASSO regression model	PET/CT radiomics	*ALK* rearrangement	adNSCLC, stage I-IV	N = 367	N = 159	0.88
[51]	Shao et al., 2022	Multi-label multi-task deep learning (MMDL) system	CT radiomics	Various driver mutations; PD-L1 tumor proportion score (TPS) ≥ 50%	NSCLC	N = 876	N = 110	0.796 (*EGFR*); 0.867 (*ALK*); 0.680 (*BRAF*); 0.816 (*KRAS*); 0.912 (PD-L1 TPS ≥ 50%)
[52]	Jiang et al., 2021	ML-based models, including random forest, decision tree, LG, AdaBoost, Gaussian process, and support vector machine	CT radiomics	PD-L1 expression	Resected adNSCLC	N = 91	N = 34	0.85 (CT-based hand-crafted radiomic signature); 0.61 (radiomics-nomogram model); 0.38 (clinical model)
[53]	Tian et al., 2021	Deep convolutional neural network	CT radiomics	PD-L1 TPS ≥ 50%	NSCLC, stage IIIB-IV	N = 750	N = 96	0.78 (training cohort); 0.71 (validation cohort); 0.76 (test cohort)
[54]	Wang et al., 2022	DL	CT radiomics	PD-L1 expression	NSCLC	N = 908	N = 227	0.950 (TPS < 1%); 0.934 (TPS: 1–49%); 0.946 (TPS ≥ 50%)
[55]	Mu et al., 2021	Small-residual-convolutional-network (SResCNN)	PET/CT radiomics	PD-L1 expression; Response to ICI	NSCLC, stage I-IV from 5 cohorts (bi-institutional; China and Florida)	N = 284 (PD-L1 expression); N = 177 (Response to ICI)	N = 116 (PD-L1 expression); N = 35 (Response to ICI)	0.82 (PD-L1 expression); c-indexes of 0.7–0.87 for the combination of Deep-Learned score (DLS) with clinical characteristics (response to ICI)
[56]	Jiang et al., 2020	LASSO regression model	CT, PET, and PET/CT radiomics	PD-L1 expression	NSCLC, stage I-IV	N = 266	N = 133	0.97, 0.61, and 0.97 (PD-L1 > 1%, CT, PET, and PET/CT radiomics, respectively); 0.80, 0.65, and 0.77 (PD-L1 > 50%, CT, PET, and PET/CT radiomics, respectively)
[57]	Trebeschi et al., 2019	Gene set enrichment analysis (GSEA)	CT radiomics	Response to PD-1 ICI	Advanced NSCLC under anti-PD1 therapy	N = 123	N = 262	0.79
[58]	Gong et al., 2022	Support vector machine (SVM) classifier	Delta radiomics	Response to ICI	NSCLC, clinical stage III–IV, under immunotherapy alone	N = 93	N = 131	0.82–0.87
[59]	Ramella et al., 2018	Random forest classifier	CT radiomics	Response to concurrent chemoradiation (cCRT)	NSCLC, stage III, treated with cCRT	N = 91	N/A	0.82
[60]	Sun et al., 2018	Linear elastic-net ML model	CT radiomics	CD8 gene expression; tumor immune phenotype	LC	N = 30	N = 119 (CD8 gene expression); N = 100 (tumor immune phenotype)	0.67 (CD8 gene expression); 0.76 (tumor immune phenotype)
[61]	Sun et al., 2020	N/A	CT radiomics	CD8 gene expression as a predictive biomarker of response to ICI + RT	Advanced NSCLC	N = 14	N/A	0.63
[62]	Mu et al., 2020	LASSO regression model	PET/CT radiomics	TME image features to predict response to ICI	NSCLC, stage IIIB–IV	N = 99	N = 95	0.86 (training cohort); 0.83 (retrospective test cohort); 0.81 (prospective test cohort)

**Table 2:** Selected studies of AI-based pathomics in NSCLC treatment.

Reference	Author, Year	AI-Based Methodology	Model Features	Target Variable	Dataset Source	Training Cohort	Validation Cohort	Best Performance
[76]	Coudray et al., 2018	Deep convolutional neural network (CNN)	Pathomics	Molecular classification	The Cancer Genome Atlas (TCGA) database	N = 1144 whole slide images	N = 490 whole slide images	0.97 (adNSCLC, sqNSCLC, and healthy tissue discrimination); 0.733–0.856 (molecular classification)
[77]	Mayer et al., 2022	Advanced CNN	Pathomics	*ALK* and *ROS1* fusion identification	NSCLC patients (single institution)	N = 162	N = 72	Sensitivity: 100% (for both genes); Specificity: 100% (for ALK fusion) and 98.6% (for ROS1 fusion)
[78]	Ren et al., 2023	DL	Pathomics	Diagnosis and gene alteration prediction	Pleural effusion cell block whole-slide images (single institution)	N = 410	N/A	0.932 (diagnosis); 0.869 (*ALK* fusion); 0.804 (*KRAS* mutation); 0.644 (*EGFR* mutation); 0.774 (no alterations)
[79]	Rakaee et al., 2023	QuPath v.0.2.3 (supervised ML algorithm)	Pathomics	Tumor-infiltrating lymphocytes (TILs), TMB, and PD-L1 as predictive biomarkers of ICI	ICI-treated, advanced-stage NSCLC	N = 284	N = 97	0.70 (PD-L1/TMB); 0.56 (PD-L1/TILs); 0.52 (PD-L1); 0.77 (TILs, in PD-L1 TPS < 1%); 0.65 (TMB, in PD-L1 TPS < 1%)
[80]	Hondelink et al., 2022	4 separate CNNs	Pathomics	PD-L1 expression	NSCLC	N = 60	N = 139	79% concordance with the reference score
[81]	Nibid et al., 2023	5 separate CNNs	Pathomics	Response to cCRT	NSCLC, stage IIIA/IIIB under cCRT	N = 33	N = 2	TPr = 0.75; TNr = 90.1
[82]	Lin et al., 2022	ML	Pathomics	CD3+ T-cell and CD8+ T-cell density in TME and its prognostic value	NSCLC patients who underwent upfront surgery	N = 145	N = 180	DFS HR: 0.57 for the high l-score (*p* = 0.022)
